# Predicting lncRNA–Protein Interactions With miRNAs as Mediators in a Heterogeneous Network Model

**DOI:** 10.3389/fgene.2019.01341

**Published:** 2020-01-22

**Authors:** Yuan-Ke Zhou, Zi-Ang Shen, Han Yu, Tao Luo, Yang Gao, Pu-Feng Du

**Affiliations:** ^1^ College of Intelligence and Computing, Tianjin University, Tianjin, China; ^2^ School of Medicine, Nankai University, Tianjin, China

**Keywords:** heterogeneous network, lncRNA–protein interaction, lncRNA–miRNA interaction, miRNA–protein interaction, network similarity

## Abstract

Long non-coding RNAs (lncRNAs) play important roles in various biological processes, where lncRNA–protein interactions are usually involved. Therefore, identifying lncRNA–protein interactions is of great significance to understand the molecular functions of lncRNAs. Since the experiments to identify lncRNA–protein interactions are always costly and time consuming, computational methods are developed as alternative approaches. However, existing lncRNA–protein interaction predictors usually require prior knowledge of lncRNA–protein interactions with experimental evidences. Their performances are limited due to the number of known lncRNA–protein interactions. In this paper, we explored a novel way to predict lncRNA–protein interactions without direct prior knowledge. MiRNAs were picked up as mediators to estimate potential interactions between lncRNAs and proteins. By validating our results based on known lncRNA–protein interactions, our method achieved an AUROC (Area Under Receiver Operating Curve) of 0.821, which is comparable to the state-of-the-art methods. Moreover, our method achieved an improved AUROC of 0.852 by further expanding the training dataset. We believe that our method can be a useful supplement to the existing methods, as it provides an alternative way to estimate lncRNA–protein interactions in a heterogeneous network without direct prior knowledge. All data and codes of this work can be downloaded from GitHub (https://github.com/zyk2118216069/LncRNA-protein-interactions-prediction).

## Introduction

Non-coding RNAs (ncRNAs) refer to RNAs that do not encode proteins. These genes were once considered as “junk DNAs” or “dark matters” in the genome (Schaukowitch and Kim, 2014). However, over the last few years, more and more functioning ncRNAs have been discovered, such as ribosomal RNAs(rRNA), ribozymes, transfer RNAs (tRNA), small nuclear RNAs (snRNAs), small nucleolar RNAs (snoRNAs), micro RNAs (miRNAs), long noncoding RNAs (lncRNAs), and many others (Peculis, 2000; Henras et al., 2004; Okamura and Lai, 2008; Kung et al., 2013). All these ncRNAs can influence biological progress on various levels (Louro et al., 2009).

Long non-coding RNAs are ncRNAs with a length larger than 200 nt (Kapranov et al., 2007). Experiments show that lncRNA–protein interactions play important roles in many biological processes, such as splicing, polyadenylation, and translation (Singh, 2002; Lukong et al., 2008; Kishore et al., 2010; Licatalosi and Darnell, 2010). Therefore, studying interactions between lncRNAs and proteins makes great sense for us to understand a wide variety of biological processes.

Although we can now obtain RPIs (RNA–protein interactions) through large-scale experiments such as RNAcompete (Ray et al., 2009), RIP-Chip (Keene et al., 2006), HITS-CLIP (Licatalosi et al., 2008), and PAR-CLIP (Hafner et al., 2010), all these experiments are costly and time-consuming. Therefore, computational predictions have been recognized as an efficient alternative approach. Muppirala et al. (2011) proposed the RPISeq method for predicting RNA–protein interactions using only sequence information. Wang et al. (2013) extracted sequence-based features to represent each protein–RNA pair and used naive-Bayes classifier to predicting protein–RNA interactions. Lu et al. (2013) introduced a new method named lncPro, which scored each RNA–protein pair by encoding RNA and protein sequences into numerical vectors. Suresh et al. (2015) presented an SVM-based method, named RPI-Pred, to predict protein–RNA interaction pairs based on their sequences and structures. Li et al. (2015) developed a heterogeneous network model (LPIHN) and a random walk with restart algorithm to predict novel lncRNA–protein interactions. Ge et al. (2016) constructed the lncRNA–protein bipartite network, and scored candidate proteins for each lncRNA based on the bipartite network projection algorithm. Yang et al. (2016) constructed another lncRNA–protein bipartite network, where the HeteSim algorithm was employed to evaluate the relevance between lncRNAs and proteins. Zheng et al. (2017) applied the HeteSim algorithm on the fusion of multiple protein–protein similarity networks to predict lncRNA–protein interactions. Hu et al. (2017) presented transformation-based semi-supervised link prediction (LPI-ETSLP) to predict lncRNA–protein interactions. Xiao et al. (2017) proposed a computational method named PLPIHS for predicting lncRNA–protein interactions using HeteSim Scores. Hu et al. (2018) presented a model named HLPI-Ensemble integrated three mainstream machine learning algorithms for predicting human lncRNA–protein interaction. Zhang et al. (2018b) combined multiple similarities and features with a feature projection ensemble learning frame to predict lncRNA–protein interactions. Zhang et al. (2018a) proposed a linear neighborhood propagation method (LPLNP) to calculate the linear neighborhood similarity of lncRNA–protein interactions. Zhang et al. (2019c) proposed the KATZLGO method to predict lncRNA–protein interactions based on the KATZ measure, which utilize the information of all paths between pair of nodes.

All existing methods rely on known lncRNA–protein interactions to construct the predictor. However, the number of experimentally verified lncRNA–protein interactions is limited, which affects the prediction performances of all existing methods. To expand the spectrum of predictable lncRNA–protein interactions, we took miRNAs as intermediates in predicting lncRNA–protein interactions.

MiRNAs are short RNA molecules with a length of 19 to 25 nucleotides (Lu and Rothenberg, 2018). Some miRNAs can regulate both lncRNAs and proteins. For example, PTEN (Phosphatase and TENsin homolog) is a kind of tumor suppressor gene, which is critical for maintaining cellular homeostasis (Poliseno et al., 2010). The miR-21 regulates the translation process of PTEN (Zhang et al., 2010), as well as the expression of PTENpg1, which is transcribed from PTEN pseudogene as an lncRNA (Yu et al., 2014). Meanwhile, the PTENpg1 alpha isoform affects the transcription process of PTEN by competing transcription factors (Johnsson et al., 2013). We assumed that this triangular regulation network can be common in the gene regulation system. To validate this assumption, we collected lncRNA–miRNA interactions and protein–miRNA interactions from the RAID v2.0 database. We found that the lncRNA–protein interactions are significantly enriched in the set of lncRNAs and proteins that are sharing a common set of interacting miRNAs (chi-square test, p-value < 10^-16^).

In the light of this observation, miRNAs were taken as mediators to predict novel lncRNA–protein interactions in this work. Both lncRNA–miRNA interactions and miRNA–protein interactions were considered as the basis to predict lncRNA–protein interactions. In the cause of improving our prediction performance, the similarity of lncRNAs and proteins was calculated in various aspects, which is based on the assumption that similar lncRNAs or proteins tend to have similar interactions. Our methods provide a way to explore novel lncRNA–protein interactions without prior knowledge of direct lncRNA–protein interactions. Since existing methods always require direct lncRNA–protein interactions as training data, our method may provide a useful supplement to the state-of-the-arts methods.

## Materials and Methods

### Dataset Curation

Biomolecule interactions have become a hot research topic in computational biology. RAID v2.0 is a large database for biomolecule interaction information, which contains more than 5.27 million RNA-associated interactions, including over 4 million RNA–RNA interactions and 1.2 million RNA–protein interactions, involving nearly 130,000 genes across 60 species (Yi et al., 2017). We downloaded the protein–miRNA interactions and lncRNA–miRNA interactions as our training dataset from this database. LncRNA–protein interactions were also obtained as our independent testing dataset simultaneously.

We downloaded 2,862 lncRNA–miRNA interactions and 2,521 protein–miRNAs interactions, which are all experimentally verified, from the RAID v2.0 database (Yi et al., 2017). In order to ensure that each lncRNA has a protein linked to it *via* a miRNA, and vice versa, common miRNAs were extracted from these interactions. Altogether 360 miRNAs were included in our dataset. Subsequently, the lncRNA–miRNA interactions and the protein–miRNA interactions were selected according to the common interacting miRNAs. We kept 1,356 lncRNA–miRNA interactions and 1,156 protein–miRNA interactions in our dataset. These interactions are among 331 lncRNAs, 360 miRNAs, and 103 proteins. The sequences of lncRNAs and proteins were obtained from NCBI Gene database (Brown et al., 2015) and the Uniprot database (The UniProt Consortium, 2017), respectively. For those lncRNAs, which cannot be found in the NCBI Gene database, the sequence was retrieved from the Ensemble database (Hunt et al., 2018).

In order to evaluate the performance of our predictive model, we obtained experimentally verified lncRNA–protein direct interactions from the RAID v2.0 database according to the lncRNAs and proteins in our dataset. Subsequently 1,925 lncRNA–protein interactions were chosen as our independent testing dataset, which are formed by 268 lncRNAs and 58 proteins. The interactions from the RAID database are listed in the **Supplementary Materials**(**Table S1**, **Table S2** and **Table S3**).

### Similarity Measures

Previous studies (Gong et al., 2019; Zhang et al., 2019a; Zhang et al., 2019b) have demonstrated the usefulness of similarities for network models. For convenience, let *L* be the set of lncRNAs, *M* the set of miRNAs, and *P* the set of proteins, e.g. *L* = {*l*_1_, *l*_2_, …, *l*_x_}, *M* = {*m*_1_, *m*_2_, …, *m*_y_} and *P* = {*p*_1_, *p*_2_,…, *p*_z_}, where *x* denotes the number of different lncRNAs, *y* the number of common miRNAs, and *z* the number of different proteins.

**Figure 1:**
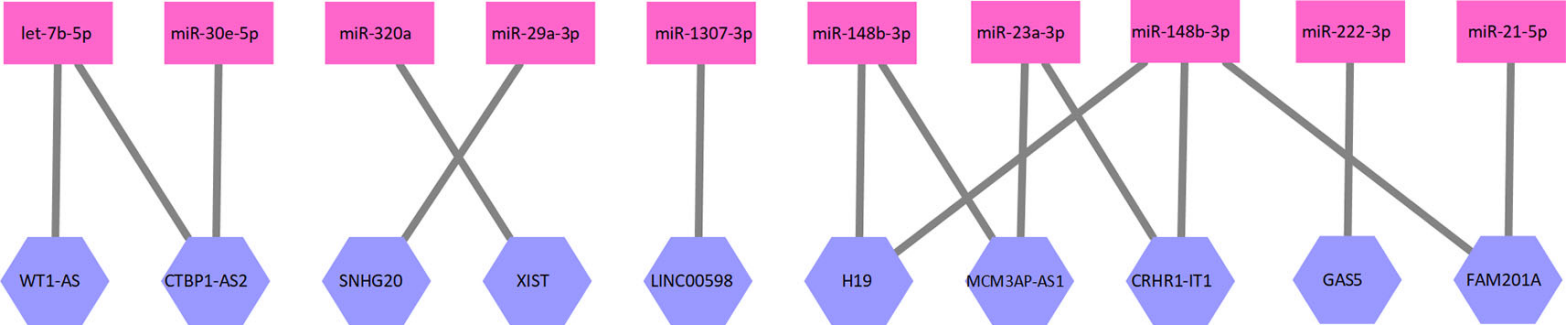
A part of the lncRNA–miRNA interaction network. Ten lncRNAs and 10 miRNAs formed this part of the interaction network. The network is a bipartite graph. One lncRNA can interact with multiple miRNA and vice versa.

The lncRNA–miRNA interaction network can be represented using a bipartite graph *G*_1_, as follows:

(1) $$G_{1}=\left(L,M,E_{1}\right),$$

where *E*_1_ is the set of edges in this bipartite graph, and *L* and *M* as defined above. Each edge in *E*_1_ represents an interaction between one lncRNA and one miRNA. A part of the lncRNA–miRNA interaction network is illustrated as **Figure 1**.

**Figure 2:**
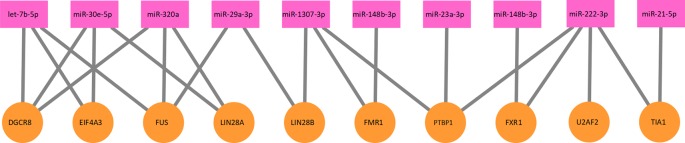
A part of the protein–miRNA interaction network. Ten proteins and 10 miRNAs formed this part of the interaction network. The network is a bipartite graph. One protein can interact with multiple miRNA and vice versa.

Similarly, we used another bipartite graph *G*_2_ to represent the protein–miRNA interaction network, as follows:

(2) $$G_{2}=\left(P,M,E_{2}\right),$$

where *E*_2_ is the edge set of the protein–miRNA interaction network, and *P* and *M* as defined above. Each protein–miRNA interaction corresponds to an edge in *E*_2_. A part of the protein–miRNA interaction network is illustrated as **Figure 2**.

With the definition of two bipartite graphs, similarities between lncRNAs or proteins were both calculated in three different ways, which are elaborated in the following sections, respectively.

#### Network Similarity

For a given miRNA, *m_k_*∈ *M* (*k* = 1, 2,...., *y*), we define the set of its interacting lncRNAs as *L*(*m_k_*), which is a subset of *L*:

(3) $$L\left(m_{k}\right)=\left\{l|(l,m_{k})\in E_{1},m_{k}\in M,l\in L\right\}.$$

We also define *P*(*m_k_*), which is a subset of *P*, as follows:

(4) $$P\left(m_{k}\right)=\left\{p|\left(p,m_{k}\right)\in E_{2},m_{k}\in M,p\in P\right\}.$$

For miRNAs in *M*, of which the network contribution in the lncRNA–miRNA interaction network or the protein–miRNA interaction network can be calculated respectively as follows:

(5) $$c_{1}\left(m_{k}\right)=-\ln\left(\left|L\left(m_{k}\right)\right|/\sum_{k=1}^{y}\left|L\left(m_{k}\right)\right|\right),\;\text{and}$$

(6) $$c_{2}\left(m_{k}\right)=-\ln\left(\left|P\left(m_{k}\right)\right|/\sum_{k=1}^{y}\left|P\left(m_{k}\right)\right|\right),$$

where *c*_1_(*m_k_*) is the network contribution of miRNA *m_k_* in the lncRNA–miRNA interaction network, *c*_2_(*m_k_*) the network contribution of miRNA *m_k_* in the protein–miRNA interaction network, and |.| the cardinal operator on a set.

For convenience, *M*(*l_i_*) and *M*(*p_j_*), which are both subsets of *M*, are defined as follows:

(7) $$M\left(l_{i}\right)=\left\{m|\left(m,l_{i}\right)\in E_{1},l_{i}\in L,m\in M\right\},\;and$$

(8) $$M\left(p_{j}\right)=\left\{m|\left(m,p_{j}\right)\in E_{2},p_{j}\in P,m\in M\right\},$$

where *M*(*l_i_*) and *M*(*p_j_*) represent the set of miRNAs that interact with a given lncRNA or a given protein.

With all definition above, the network similarity between two lncRNAs *l_u_* and *l_v_* (*u*, *v* = 1, 2,…, *x*) can be defined as follows:

(9) $$n_{1}\left(l_{u},l_{v}\right)=\sum_{m_{k}\in M\left(l_{u}\right)\cap M\left(l_{v}\right)}c_{1}\left(m_{k}\right),$$

where *n*_1_(*l_u_*, *l_v_*) is the network similarity between *l_u_* and *l_v_*.

Similarly, given two proteins, *p_u_* and *p_v_*, the network similarity between *p_u_* and *p_v_* can be defined as follows:

(10) $$n_{2}\left(p_{u},p_{v}\right)=\sum_{m_{k}\in M\left(p_{u}\right)\cap M\left(p_{v}\right)}c_{2}\left(m_{k}\right),$$

where *n*_2_(*p_u_*, *p_v_*) is the network similarity between *p_u_* and *p_v_*.

#### Sequence Similarity

The sequence similarity was calculated by the Smith-Waterman algorithm. Given two lncRNAs, the sequence similarity between two lncRNA sequences is defined as follows:

(11) $$e_{1}(l_{u},l_{v})=\frac{w\left(l_{u},l_{v}\right)}{\left|l_{u}\right|+\left|l_{v}\right|},$$

where *e*_1_(*l_u_*, *l_v_*) is the sequence similarity, *w*(*l_u_*, *l_v_*) the Smith-Waterman score between *l_u_* and *l_v_*, and |*l_u_*| and |*l_v_*| the length of the lncRNA *l_u_* and *l_v_*, respectively.

Given two proteins, the sequence similarity between two protein sequences is defined similarly as follows:

(12) $$e_{2}\left(p_{u},p_{v}\right)=\frac{w\left(p_{u},p_{v}\right)}{\left|p_{u}\right|+\left|p_{v}\right|},$$

where *e*_2_(*p_u_*, *p_v_*) is the sequence similarity, and *w*(*p_u_*, *p_v_*), |*p_u_*|, and |*p_v_*| the length of the protein *p_u_* and *p_v_*, respectively.

#### Statistical Feature Similarity

Pseudo-amino acid composition (PseAAC), which was proposed by Chou in 2001 (Chou, 2001), has been widely applied in all branches of computational and functional proteomics (Chou, 2011; Chou, 2015). Pseudo-k nucleotides composition (PseKNC), which is a major advancement of the PseAAC concept in analyzing nucleotide sequences, has been introduced recently (Chen et al., 2014). Because of its simplicity and effectiveness, the PseKNC methods quickly penetrate into all major topics in functional genomics, in both genome and transcriptome levels (Chen et al., 2015a; Chen et al., 2015b). The computational procedures for PseAAC and PseKNC have been elaborated in many literatures (Chou, 2011; Chen et al., 2013; Qiu et al., 2017) and some recent reviews (Chen et al., 2015a; Zhao et al., 2018).

In this work, we employed pseudo di-nucleotide composition (PseDNC), which is a special form of PseKNC when k = 2, to represent lncRNA sequences, and PseAAC for protein sequencesFor simplicity, we do not describe the computational details of the PseDNC and PseAAC algorithms here. We only describe how we apply PseDNC and PseAAC in our work.

Given a lncRNA, its PseDNC representation can be described as a numerical vector with 16+λ dimensions as follows:

(13) $$V_{1}\left(l_{i}|\lambda ,\omega_{1},H\right)={\left[\begin{array}{cccccccc} d_{1} & d_{2} & \cdots & d_{16} & d_{16+1} & d_{16+2} & \cdots & d_{16+\lambda } \end{array}\right]}^{T},$$

where **V**_1_(*l_i_* | *λ*, *ω*_1_, *H*) is the PseDNC representation of *l_i_*, *λ* and *ω*_1_ two parameters in computing the PseDNC representation, and *H* a set of di-nucleotide physicochemical properties that are applied in computing the PseDNC representations.

The similarity between two lncRNAs can be defined as follows:

(14) $$f_{1}\left(l_{u},l_{v}\right)=1/{\left\|V_{1}\left(l_{u}|\lambda ,\omega_{1},H\right)-V_{1}\left(l_{v}|\lambda ,\omega_{1},H\right)\right\|}^{2},$$

where *f*_1_(*l_u_*, *l_v_*) is the feature similarity between two lncRNAs, and ||.|| the operator that takes the length of a vector.

Similarly, given a protein, its PseAAC representation can be described as a numerical vector with 20 + τ dimensions as follows:

(15) $$V_{2}\left(p_{j}|\tau ,\omega_{2},H\right)={\left[\begin{array}{cccccccc} r_{1} & r_{2} & \cdots & r_{20} & r_{20+1} & r_{20+2} & \cdots & r_{20+\tau } \end{array}\right]}^{T},$$

where **V**_2_(*p_j_* | *τ*, *ω*_2_, *H*) is the PseAAC representation of *l_j_*, *τ* and *ω*_2_ two parameters in computing the PseAAC representation, and *H* a set of amino acid physicochemical properties that are used in computing the PseAAC representations.

The similarity between two proteins can be defined as follows:

(16) $$f_{2}\left(p_{u},p_{v}\right)=1/{\left\|V_{2}\left(p_{u}|\tau ,\omega_{2},H\right)-V_{2}\left(p_{v}|\tau ,\omega_{2},H\right)\right\|}^{2},$$

where *f*_2_(*p_u_*, *p_v_*) is the feature similarity between two proteins.

We utilized online webserver Pse-In-One (Liu et al., 2015) to generate PseDNC and PseAAC in our work.

### Heterogeneous Network Model

**Figure 3:**
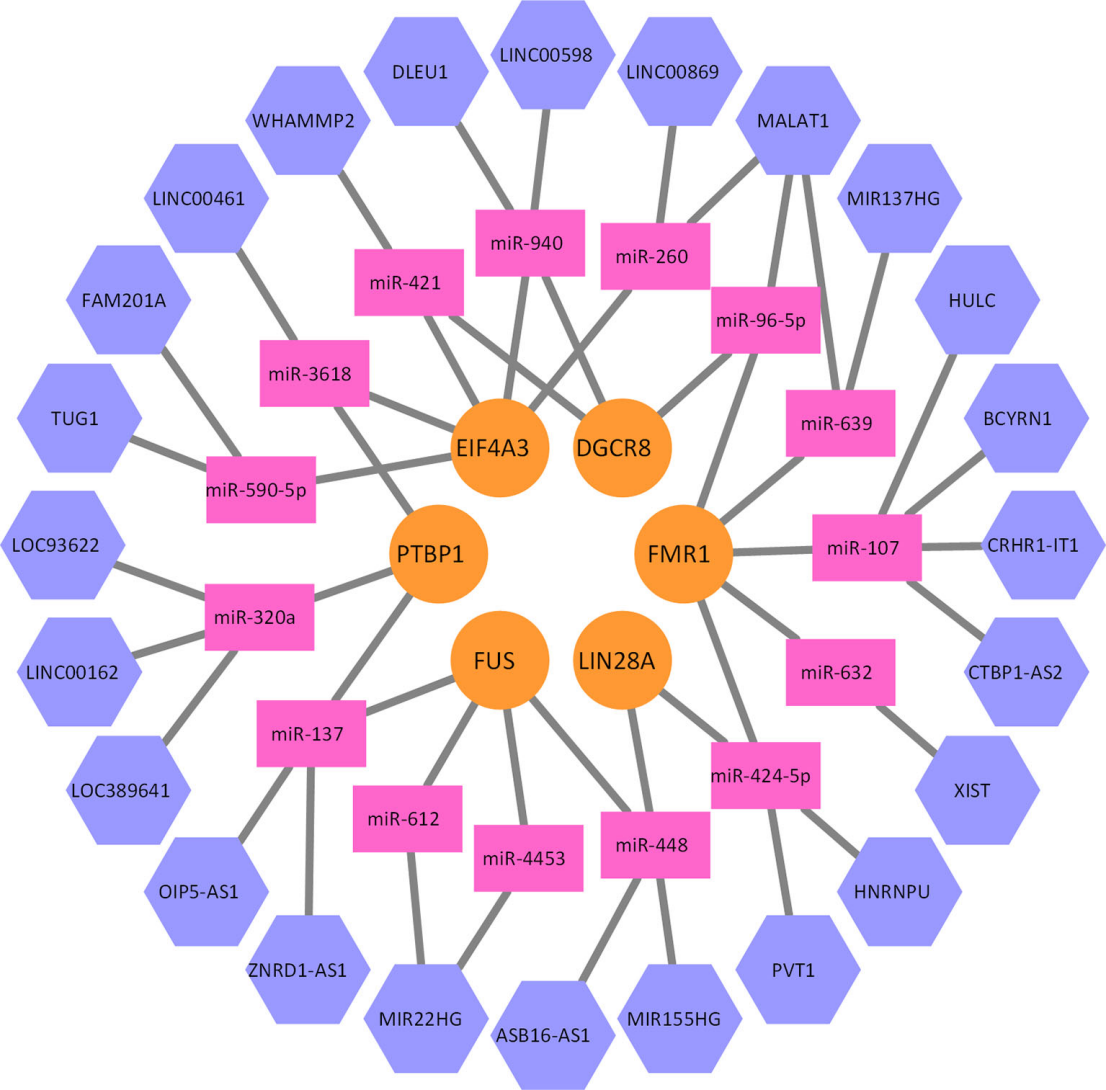
A part of the lncRNA–miRNA–protein association network. Five proteins, 15 miRNAs, and 24 lncRNAs formed this part of the interaction network. Every miRNA can interact with multiple lncRNAs, and multiple proteins as well.

By integrating the bipartite graph *G*_1_ and *G*_2_, we can construct a heterogeneous network model, where lncRNAs, miRNAs, and proteins are connected together. A part of this network is illustrated as **Figure 3**. Given a lncRNA *l_i_* and a protein *p_j_*, a whole network correlation that is brought by the *m_k_* can be defined as follows:

(17) $$t\left(m_{k}\right)=\left|L\left(m_{k}\right)\right|+\left|P\left(m_{k}\right)\right|.$$

The whole network correlation function between lncRNA *l_i_* and protein *p_j_* can be defined as follows:

(18) $$k\left(l_{i},p_{j}\right)=-\sum_{m_{k}\in M\left(l_{i}\right)\cap M\left(p_{j}\right)}\ln\left(t\left(m_{k}\right)/\sum_{k=1}^{y}t\left(m_{k}\right)\right),$$

The whole network correlation matrix can be established as **K** *=* {*k*(*l_i_*, *p_j_*)}, *i* = 1, 2,.., *x* and *j* = 1, 2,…, *z*.

For two given lncRNAs, the similarity between them can be noted as *s*_1_(*l_u_*, *l_v_*), where *l_u_* and *l_v_* are two lncRNAs. Similarly, for two given proteins, the similarity between them can be noted as *s*_2_(*p_u_*, *p_v_*). The similarity between two lncRNAs or two proteins can be measured in various aspects, which have been elaborated in the above section.

The similarity matrix for lncRNAs and proteins can be established as **S**_1__=_ {*s*_1_(*l_u_*, *l_v_*)}, *u*, *v* = 1, 2,…, *x* and **S**_2_ = {*s*_2_(*p_u_*, *p_v_*)}, *u*, *v* = 1, 2,…, *z*, respectively. We normalize the values in matrix **S**_1_ and **S**_2_ as follows:

(19) $$q_{1}\left(l_{u},l_{v}\right)=\{\begin{array}{cc} \frac{s_{1}\left(l_{u},l_{v}\right)}{\sum_{v=1}^{x}s_{1}\left(l_{u},l_{v}\right)} & u\neq v\\ 1 & u=v \end{array}\;,u,v=1,2,\ldots ,x\;\text{and}$$

(20) $$q_{2}\left(p_{u},p_{v}\right)=\{\begin{array}{cc} \frac{s_{2}\left(p_{u},p_{v}\right)}{\sum_{v=1}^{z}s_{2}\left(p_{u},p_{v}\right)} & u\neq v\\ 1 & u=v \end{array},u,v=1,2,\ldots ,z$$

where *q*_1_(*l_u_*, *l_v_*) and *q*_2_(*l_u_*, *l_v_*) are normalized value in **S**_1_ and **S**_2_. We note the normalized matrix as **Q**_1_ and **Q**_2_ respectively, where **Q**_1__=_ {*q*_1_(*l_u_*, *l_v_*)}, *u*, *v* = 1, 2,…, *x* and **Q**_2_ = {*q*_2_(*p_u_*, *p_v_*)}, *u*, *v* = 1, 2,…, *z*.

With all above definitions, we can establish the final scoring matrix as follows:

(21) $$W=Q_{1}KQ_{2}$$

The prediction of lncRNA–protein interactions is made based on the scores in **W**. If a value in **W** were larger than a given threshold, the corresponding lncRNA and protein would be predicted to interact. Otherwise, no interaction would be predicted.

**Figure 4:**
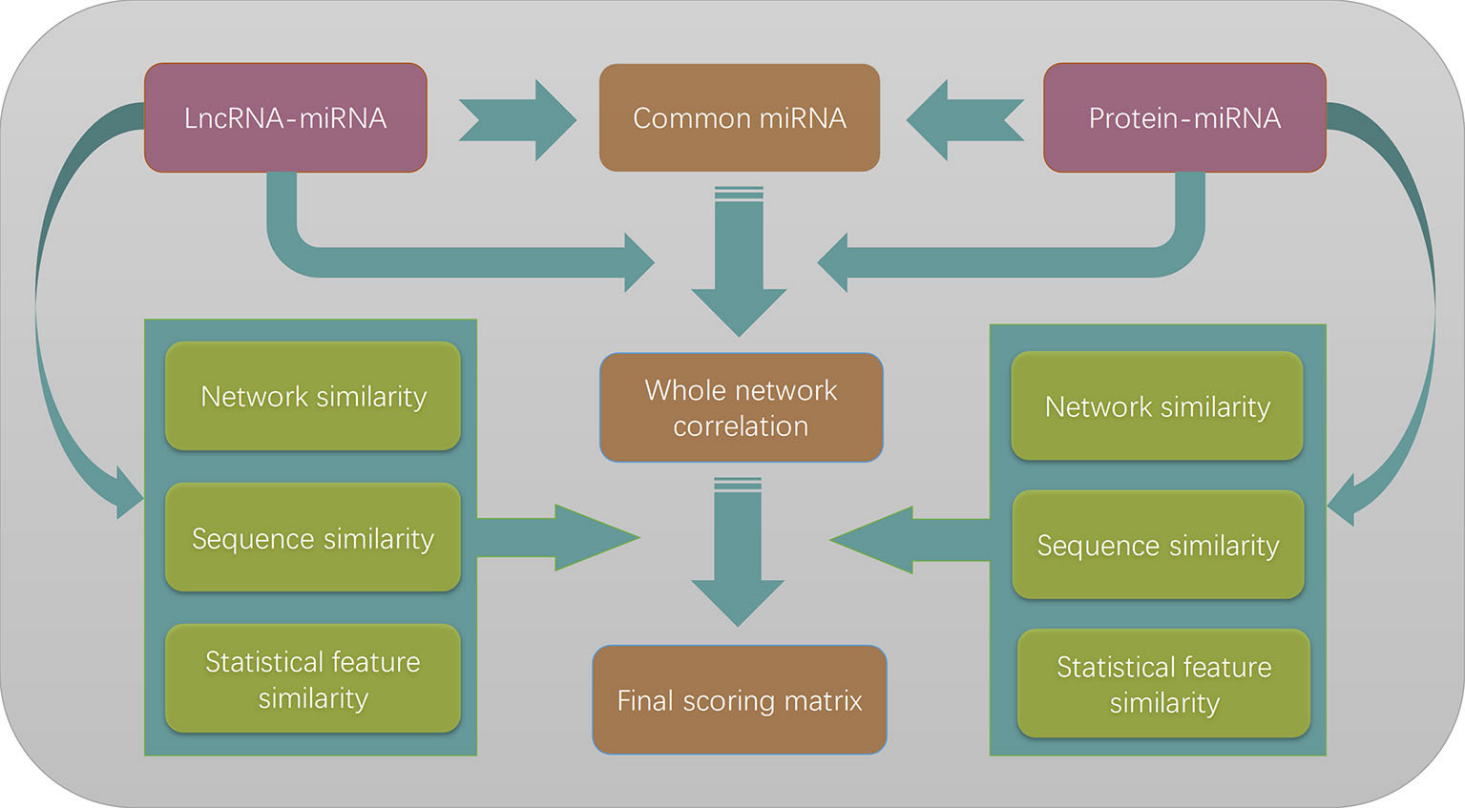
The flowchart of the entire work. The lncRNA–miRNA interactions and protein–miRNA interactions are merged to form a heterogeneous network model according to the commonly shared miRNAs in two different types of interactions. Three different similarity measures are combined with the whole network correlation matrix to form nine different scoring matrices. By optimizing the prediction performances, we finally choose to use network similarity measures for both lncRNAs and proteins.

The whole flowchart of our method is illustrated in **Figure 4**. Three different similarity measures were applied to lncRNAs and proteins, respectively. Since they can be chosen independently to each other, there are nine different combinations of the similarity choices

### Performance Evaluation

Given a threshold, a set of lncRNA–protein interactions can be predicted from the matrix **W**. By comparing this set against the testing dataset, the number of true positives (*TP*), true negatives (*TN*), false positives (*FP*), and false negatives (*FN*) can be obtained, respectively. Five statistical measures can be calculated as follows:

(22) $$TPR=\frac{TP}{TP+FN},$$

(23) $$FPR=\frac{FP}{FP+TN},$$

(24) $$Pre=\frac{TP}{TP+FP},$$

(25) $$Rec=\frac{TP}{TP+FN},\;and$$

(26) $$Acc=\frac{TP+TN}{TP+TN+FP+FN},$$

where *TPR* is for true positive rate, *FPR* for false positive rate, *Pre* for precision, *Rec* for recall, and *Acc* for accuracy.

By varying the threshold from the maximum value to the minimal value in **W**, a receiver operating curve (ROC) can be plotted using the TPR and the FPR values. In the meantime, a precision-recall (PR) curve can be obtained using the precision and the recall values. Due to the nature that the negatives are far more than the positives in the current topic, the area under the ROC (AUROC) and the area under the PR curve (AUPR) are used both as primary performance measures of our method.

### Parameter Calibrations

In our work, there are parameters when the PseDNC and the PseAAC sequence representations are generated. We used a grid search strategy to find the optimal parameters in the PseDNC and PseAAC. The parameter *λ* varies from 10 to 20 with a step of 1, *ω*_1_ from 0.1 to 1 with a step of 0.1, *τ* from 10 to 20 with a step of 1, and *ω*_2_ from 0.05 to 0.5 with a step of 0.05. We finally choose *λ* = 10, *ω*_1_ = 0.1, *τ* = 11 and *ω*_2_ = 0.5. The physicochemical properties in the PseDNC are Rise, Tilt, Twist, Slide, Shift, and Roll, which are defined in Pse-In-One (Liu et al., 2015). The physicochemical properties in the PseAAC are HOPT810101, JOND750101, ZIMJ680104, KRIW790103, TAKK010101, ROSM880104, BLAS910101, and KRIW790101, which are all defined in AAIndex (Kawashima et al., 2008).

## Results and Discussion

### Performance Analysis

**Figure 5:**
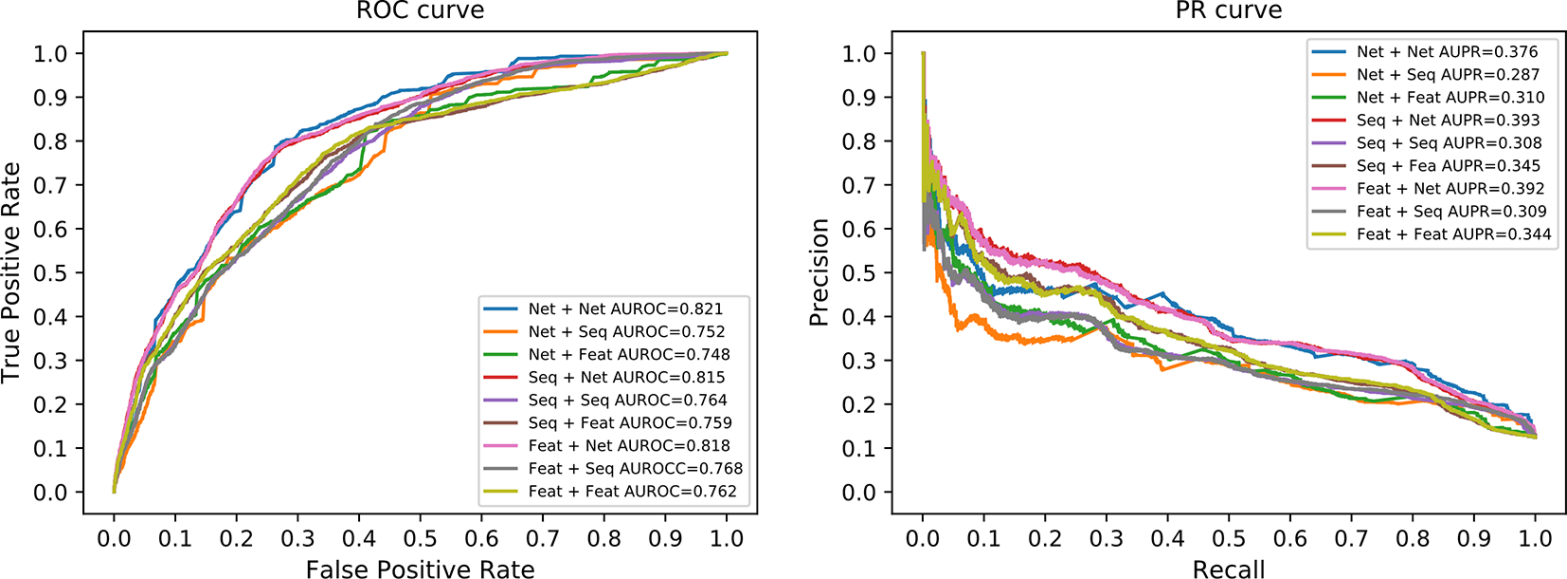
ROC and PR curves of nine similarity combinations. The horizontal axis in ROC (left panel) is for FPR and the vertical axis for TPR. The horizontal axis in PR curve (right panel) is for recall and vertical axis for precision. Net is for network similarity. Seq is for sequence similarity. Feat is for statistical feature similarity. The first part in the legend is for similarity measures of lncRNAs and the latter part for proteins. For example, the Net+Net means that we used network similarity for lncRNAs and proteins. The Net+Seq means that we used network similarity for lncRNAs and sequence similarity for proteins.

We compared the prediction performance under different combinations of similarity matrices. **Figure 5** illustrates the ROC and PR curve of our method with nine different similarity combinations. The AUROC and AUPR values were collected in **Table 1**. According to these values, the prediction performance of our method is optimized when the network similarity measure was applied to both lncRNAs and proteins. Under this condition, the AUROC achieved 0.821, while the AUPR achieved 0.376. It seems like the AUPR is low. However, by analyzing the PR curve, we found that the precision is low when the recall is in the range of (0.1, 0.4). That is to say, some lncRNA–protein pairs with a high correlation score have no experimentally verified interaction between them. This may be because these interactions are not discovered yet.

**Table 1:** AUROC and AUPR of nine similarity combinations.

Similarity matrix	AUROC^a^	AUPR^b^
Net^c^ + Net	0.821	0.376
Net + Seq^d^	0.752	0.287
Net + Feat^e^	0.748	0.310
Seq + Net	0.815	0.393
Seq + Seq	0.764	0.308
Seq + Feat	0.758	0.345
Feat + Net	0.818	0.392
Feat + Seq	0.768	0.309
Feat + Feat	0.762	0.344

a AUROC, Area under receiver operating curve.

b AUPR, Area under precision-recall curve.

c Net, Network similarity.

d Seq, Sequence similarity.

e Feat, Statistical feature similarity.

Due to the nature that the negatives are far more than the positives in predicting lncRNA–protein interactions, the testing dataset is highly imbalanced. In order to provide a set of valuable prediction results in a practical application, a recommended threshold is 2.147, which will balance the *TPR* and *FPR*, and will produce 74.3% accuracy.

### Effects of the Two Similarity Matrices

**Figure 6:**
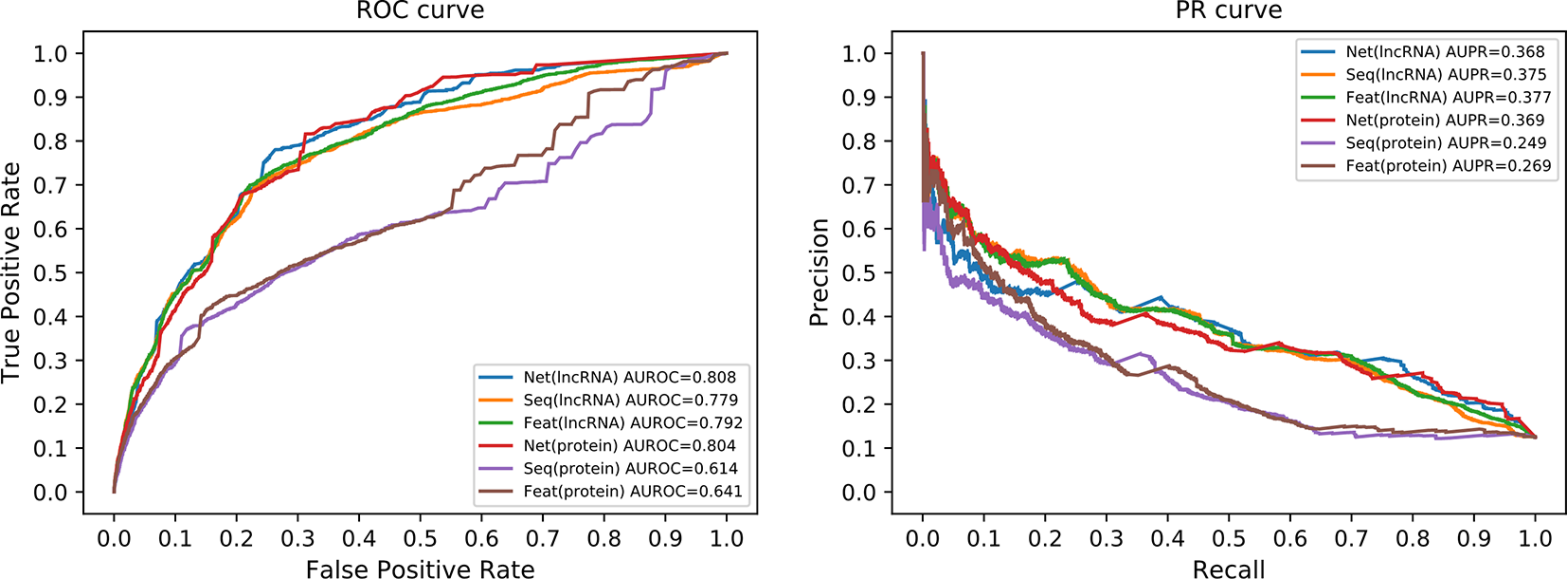
ROC curve and PR curves of single similarity matrix. Since we only use similarity matrix for lncRNAs or proteins, not the same time, we only have six different curves in each panel. The axis in both panels have the same meaning as in **Figure 5**, respectively. The “Net,” “Seq,” and “Feat” have the same meaning as the legends of **Figure 5**.

In order to analyze the effect of every single similarity matrix individually, we combined every single similarity matrix solely with the whole network correlation matrix, respectively. In other words, either **Q**_1_ or **Q**_2_ is removed from Eq (21) to see the effect of the other matrix solely. The ROC and the PR curve of all six different configurations are illustrated in **Figure 6**. The network similarity for lncRNAs performs the best among three similarities for lncRNAs. For proteins, the best similarity measure is also the network similarity. This result consists with the other results in our work. Therefore, we can safely conclude that the network similarity best suits our method. Particularly, the network similarity matrix for protein achieved a very close prediction performance to the comprehensive form of our model. Since the number of proteins and lncRNAs are imbalanced in our dataset, the number of interactions from miRNAs to proteins is far more than that to lncRNAs on average. This may be the reason that why the network similarity matrix for proteins can achieve a very promising performance solely with the whole network correlation matrix.

### Comparison With Existing Methods

**Figure 7:**
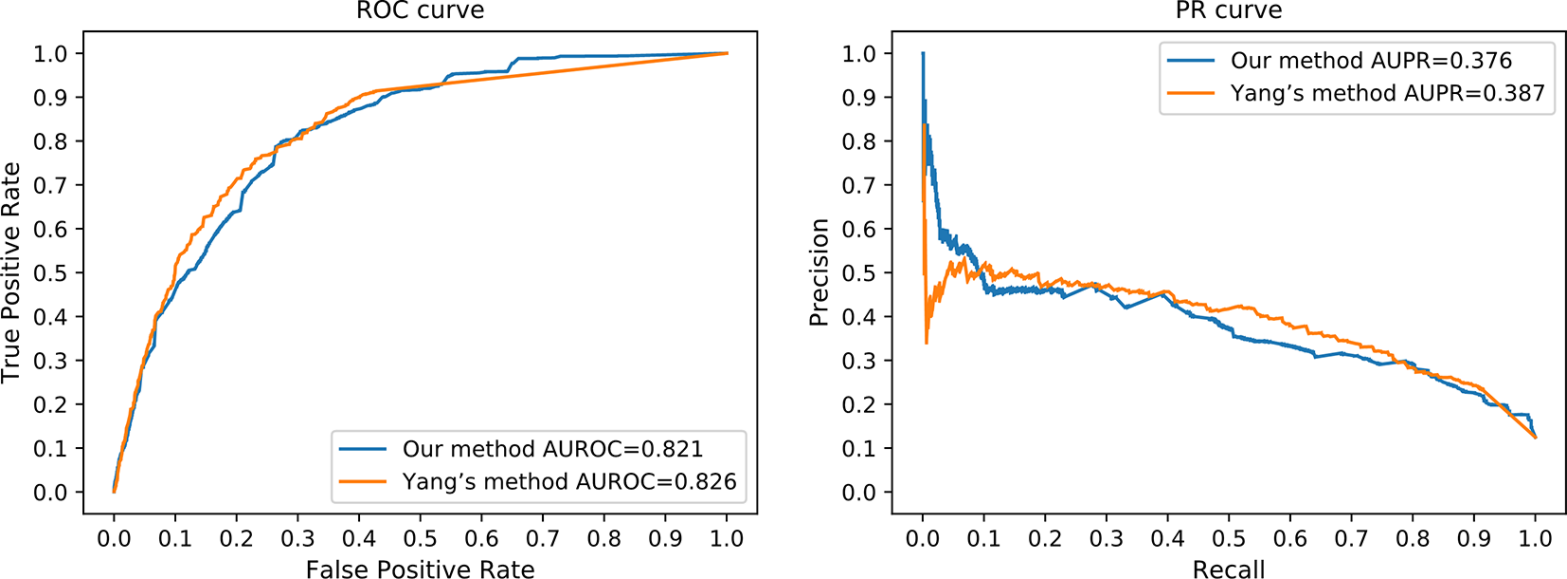
The ROC curve and the PR curve of our method and Yang's method.

HeteSim is a widely applied measure, which aims at quantifying the correlation of nodes in a heterogeneous network (Shi et al., 2014). It has been used in predicting various types of interactions and connections (Shi et al., 2014). Due to the mechanism difference between our method and existing methods, it is difficult to perform a completely fair comparison. We compared our method to Yang's work (Yang et al., 2016), where HeteSim is employed to measure the correlation between lncRNAs and proteins. In order to perform a sufficiently fair comparison, we obtained protein–protein interaction from the STRING database (Mering et al., 2003) to satisfy the requirement of Yang's work. Same testing datasets were applied to evaluate the prediction performance of Yang's work and our method simultaneously. However, due to the different mechanisms between our method and Yang's work, we tested our method using the independent testing dataset, while fivefold cross-validation was applied on Yang’s method with the same dataset. Since fivefold cross-validation may produce overestimated performance values, we believe that our method achieved a comparable performance in this comparison (**Figure 7**).

### Prediction of Novel Interactions

In order to evaluate the actual prediction effect of our method, we selected 20 interactions that are top ranked in our results. These interactions are recorded in **Table 2**. Fifteen out of 20 interactions in **Table 2** had been verified by CLIP-seq (Ule et al., 2005) in RAID v2.0. Two of the remaining five had been verified by eCLIP (Van Nostrand et al., 2016) in NPInter database (Teng et al., 2019). Since our method does not require any prior knowledge of direct lncRNA–protein interactions, and all data in our method came only from the RAID v2.0 database, our method should have a good performance. Although other three interactions are not verified, it is possible that they are undiscovered interactions under certain conditions.

**Table 2:** 20 top-ranked predictions from this work.

LncRNA^a^	Species	Protein^b^	Species	Verified?^c^
XIST	*Homo sapiens*	DGCR8	*Homo sapiens*	RAID04292086
XIST	*Homo sapiens*	EIF4A3	*Homo sapiens*	RAID05222952
BCYRN1	*Homo sapiens*	DGCR8	*Homo sapiens*	ncRI-40427659
XIST	*Homo sapiens*	LIN28A	*Homo sapiens*	RAID04539901
XIST	*Homo sapiens*	FUS	*Homo sapiens*	RAID04292231
XIST	*Homo sapiens*	DGCR8	*Homo sapiens*	RAID04639959
MALAT1	*Homo sapiens*	DGCR8	*Homo sapiens*	RAID05228988
MCM3AP-AS1	*Homo sapiens*	FUS	*Homo sapiens*	RAID04862787
MCM3AP-AS1	*Homo sapiens*	DGCR8	*Homo sapiens*	RAID04826597
MCM3AP-AS1	*Homo sapiens*	LIN28A	*Homo sapiens*	None
MCM3AP-AS1	*Homo sapiens*	LIN28B	*Homo sapiens*	ncRI-40454080
OIP5-AS1	*Homo sapiens*	DGCR8	*Homo sapiens*	RAID05191621
XIST	*Homo sapiens*	LIN28B	*Homo sapiens*	RAID05100914
CTBP1-AS2	*Homo sapiens*	FUS	*Homo sapiens*	RAID04330329
MCM3AP-AS1	*Homo sapiens*	EIF4A3	*Homo sapiens*	RAID04868241
XIST	*Homo sapiens*	FMR1	*Homo sapiens*	RAID04486531
MALAT1	*Homo sapiens*	EIF4A3	*Homo sapiens*	RAID05074375
CRHR1-IT1	*Homo sapiens*	DGCR8	*Homo sapiens*	RAID05171544
IGF2-AS	*Homo sapiens*	DGCR8	*Homo sapiens*	None
CTBP1-AS2	*Homo sapiens*	LIN28A	*Homo sapiens*	None

a lncRNA: The lncRNA names in the Gene or the Ensemble database.

b protein: The protein names in the UniProt database.

c Verified: If the direct interaction had been verified by experiment in RAID or NPInter database, this column contains the RAID and NPInter interaction identifier; otherwise “None.”

### Prediction Based on Interactions of Whole Database

**Figure 8:**
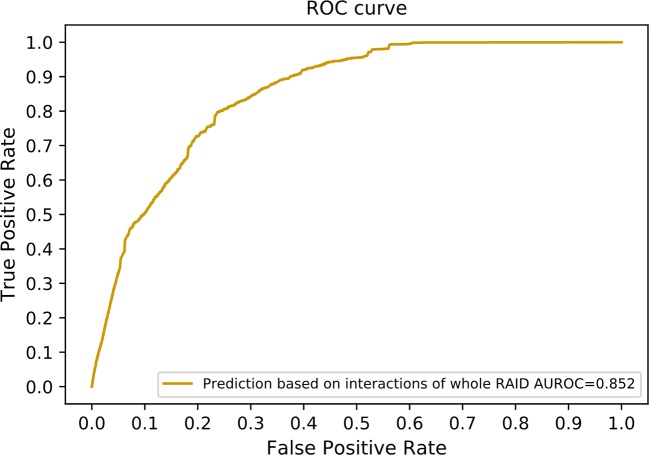
The ROC curve including interactions without experimental evidences in the RAID v2.0 database. In other words, interactions of the whole RAID database were utilized to train our model. The network similarity of both lncRNAs and proteins were selected to generate our final scoring matrix, which preformed the best in former experiment.

Since only experimentally verified interactions were obtained to compose our benchmarking dataset, a large number of predicted interactions in the RAID V2.0 database were discarded. We incorporated these predicted interactions to optimize our method. A total of 20,425 lncRNA–miRNA interactions and 1,349 protein–miRNA interactions were extracted while sharing a common set of miRNAs. These interactions are among 1,133 lncRNAs, 464 miRNAs, and 113 proteins. We also collected 2,803 lncRNA–protein interactions as our independent testing dataset. Altogether 615 lncRNAs and 65 proteins were included in this testing dataset. Our method achieved an AUROC of 0.852 on this dataset (**Figure 8**). Since our method can work with only known lncRNA–miRNA interactions and miRNA–protein interactions, it can be used as a supplement to state-of-the-art methods using direct lncRNA–protein interactions.

### Database Coverage Analysis

Due to the mechanism of our method, we restricted the lncRNA–protein interactions within those lncRNAs and proteins, which can find a sharing miRNA interactor. This restriction narrowed the profile of applicable data in the database. There are 2,862 experimentally verified lncRNA–miRNA interactions in the RAID v2.0 database, including 358 lncRNAs and 1,208 miRNAs; 1,356 lncRNA–miRNA interactions between 331 lncRNAs and 360 miRNAs were utilized in this work, accounting for 47.4%, 92.5%, and 29.8% of which in the whole database, respectively. There are 2,521 experimentally verified protein–miRNA interactions between 144 proteins and 1,032 miRNAs in the RAID v2.0 database; 1,156 protein–miRNA interactions of them were selected as our training data, composed by 103 proteins and 360 miRNAs. The protein–miRNA interactions, proteins, and miRNAs take up 45.8%, 71.5%, and 34.9% of the entire RAID database, respectively. There are 40,668 experimentally verified lncRNA–protein interactions in the RAID v2.0 database, including 3,066 lncRNAs and 10,224 proteins. The testing dataset in our work including 1,981 verified lncRNA–protein interactions between 266 lncRNAs and 58 proteins, taking up 4.87%, 8.7%, and 0.57% of which in the database, respectively. Due to the limited number of known miRNA–protein interactions and miRNA–lncRNA interactions, the coverage of proteins in the whole database is low.

We admit that this will limit the application scope of our method. However, we believe this will get better when the number of available miRNA–protein interactions is increased, because the statistical test has already shown that the lncRNA–protein interactions are significantly enriched in the set of lncRNAs and proteins that are sharing a common set of miRNAs.

## Conclusion

LncRNAs can affect biological processes from various levels. It is of great importance to study the molecular functions of lncRNAs. In the meanwhile, LncRNAs perform their role mostly by their interaction with proteins. Therefore, lncRNA–protein interaction should be studied in detail. In this paper, we proposed a method to predict lncRNA–protein interactions without prior knowledge of existing lncRNA–protein interactions. Instead, we utilized the lncRNA–miRNA interactions and the miRNA–protein interactions as the basis of our prediction. The miRNAs are used as mediators to connect the realm of lncRNAs and the realm of proteins. This is based on the hypothesis that a lncRNA and a protein may interact if they share interacting miRNAs. By quantitatively modelling the heterogeneous network that is formed by lncRNAs, miRNA, and proteins, we developed a simple, yet effective, method to predict the lncRNA–protein interactions. The best similarity measure in our method is the network similarity, which does not rely on sequence information. This gives our method a unique capability to predict lncRNA–protein interaction without comprehensive sequence information of both interactors. By comparing our predictions to the known lncRNA–protein interactions, we can conclude that our method has, at least, a comparable prediction performance to the state-of-the-art methods. Since our method does not rely on prior knowledge of lncRNA–protein interactions, it is a helpful supplement to existing methods.

## Data Availability Statement

All datasets generated for this study are included in the article/**Supplementary Material**.

## Author Contributions

Y-KZ curated the dataset, designed the algorithm, implemented the algorithm, and calibrated the parameters. Z-AS and HY performed the experiments and collected the results. TL, YG, and P-FD investigated the question, designed the whole study, conceptualized the algorithm, analyzed the results, and wrote the manuscript.

## Funding

This work was supported by the National Key R&D Program of China (2018YFC0910405); the National Natural Science Foundation of China (NSFC 61872268); and the Open Project Funding of CAS Key Lab of Network Data Science and Technology, Institute of Computing Technology, Chinese Academy of Sciences (CASNDST201705).

## Conflict of Interest

The authors declare that the research was conducted in the absence of any commercial or financial relationships that could be construed as a potential conflict of interest.

## Supplementary Material

Table S1 2862 experimentally verified lncRNA-miRNA interactions.

Table S2 2521 experimentally verified protein-miRNA interactions.

Table S3 40668 experimentally verified lncRNA-protein interactions.
